# Prognostic Impact of Blood Tumor Mutational Burden in pMMR/MSS Metastatic Colorectal Cancer Assessed by FoundationOne^®^ Liquid CDx

**DOI:** 10.3390/cancers18030515

**Published:** 2026-02-04

**Authors:** Benoist Chibaudel, Elisabeth Carola, Hamid Mekranter, Perrine Goyer, Arnaud Saget, Olivier Oberlin, Hélène Marijon, Hubert Richa, Ida Iurisci, Honorine Gervais, Nathalie Perez-Staub, Linda Dainese, Pascal Pujol, Hanah Lamallem, Clémentine Besnard, Sofya Latrache, Alain Toledano, Aimery de Gramont

**Affiliations:** 1 Department of Medical Oncology, Hôpital Franco-Britannique—Fondation Cognacq-Jay, Cancérologie Paris Ouest, 92300 Levallois-Perret, France; helene.marijon@cognacq-jay.fr (H.M.); ida.iurisci@cognacq-jay.fr (I.I.); honorine.gervais@cognacq-jay.fr (H.G.); nathalie.perez-staub@cognacq-jay.fr (N.P.-S.); 2 Department of Medical Oncology, Groupe Hospitalier du Sud de l’Oise, 60100 Creil, France; elisabeth.carola@ghpso.fr; 3 Department of Medical Oncology, Centre Hospitalier François Quesnay, 78200 Mantes La Jolie, France; abdelhamid.mekranter@ght-yvelinesnord.fr; 4 Digestive Surgery, Groupe Hospitalier Privé Ambroise Paré Hartman, 92200 Neuilly-sur-Seine, France; goyerperrine@gmail.com (P.G.); dr.asaget@gmail.com (A.S.); docteur.oberlin@gmail.com (O.O.); 5 Department of Digestive Surgery, Hôpital Franco-Britannique—Fondation Cognacq-Jay, 92300 Levallois-Perret, France; hubert.richa@gmail.com; 6 Department of Pathology and Molecular Biology, IHP Group—Paris, 92240 Malakoff, France; ld@ihp-group.fr; 7 Department of Genetic, Centre Hospitalier Universitaire de Montpellier, 34090 Montpellier, France; p-pujol@chu-montpellier.fr; 8 Department of Radiotherapy, Hartmann Oncology Radiotherapy Group, Cancérologie Paris Ouest, 92300 Levallois-Perret, France; hanah.lamallem06@gmail.com (H.L.); c.besnard@rt-hartmann.fr (C.B.); alain.toledano@gmail.com (A.T.); 9 Department of Gastroenterology, Hôpital Franco-Britannique—Fondation Cognacq-Jay, 92300 Levallois-Perret, France; sofya.latrache@cognacq-jay.fr; 10 Cancérologie Paris Ouest, 92300 Levallois-Perret, France; aimerydegramont@gmail.com

**Keywords:** colorectal cancer, circulating tumor DNA, blood tumor mutational burden, *RAS* mutation, comprehensive genomic profiling, liquid biopsy, biomarkers, prognosis, precision medicine, survival analysis

## Abstract

**Background/Objectives:** The prognostic significance of blood tumor mutational burden (bTMB) in metastatic colorectal cancer (mCRC) remains poorly defined. While tissue-based TMB has been associated with favorable outcomes in selected colorectal cancer subgroups, the clinical meaning of bTMB in real-world practice is unclear. This study evaluated the prognostic impact of bTMB measured through liquid biopsy in an unselected cohort of patients with mCRC. **Methods:** This monocentric, real-world study included 255 adult patients with pMMR/MSS mCRC who underwent routine comprehensive genomic profiling using the FoundationOne^®^ Liquid CDx assay. bTMB was quantified in mutations per megabase (mut/Mb), and patients were classified into bTMB-low and bTMB-high groups using the cohort median. The primary endpoint was overall survival (OS). Subgroup analyses, including stratification by *RAS/BRAF* mutation status, were descriptive. **Results:** The median bTMB was 5 mut/Mb. Patients in the bTMB-high group had an increased risk of death compared with those in the bTMB-low group (hazard ratio (HR) 1.88). The adverse prognostic effect for OS of high bTMB was more pronounced in patients with *RAS* mutant tumors (HR 2.32) than with *RAS/BRAF* wild-type tumors (HR 1.81), while no prognostic impact was observed in *BRAF^V600E^* mutant tumors (HR 0.90). bTMB was strongly correlated with ctDNA fraction (*p* < 0.0001). **Conclusions:** In routine clinical practice, elevated bTMB is associated with poor prognosis in pMMR/MSS mCRC, particularly in *RAS* mutant tumors. These results contrast with prior tissue-based studies and indicate that bTMB may reflect tumor burden and aggressive disease biology rather than tumor immunogenicity. Prospective studies integrating bTMB with ctDNA fraction, tumor burden metrics, and longitudinal molecular dynamics are warranted to refine its clinical utility.

## Simple Summary

Blood-based tumor mutational burden (bTMB) can be measured from circulating tumor DNA using liquid biopsy and is increasingly used in clinical oncology. However, its prognostic value in metastatic colorectal cancer (mCRC) remains unclear. In this real-world, single-center study, we analyzed 255 patients with pMMR/MSS mCRC who underwent routine genomic profiling with the FoundationOne^®^ Liquid CDx assay. We found that patients with high bTMB had significantly shorter overall survival, especially those with *RAS* mutant tumors. These results differ from previous reports based on tissue-derived TMB, which often suggested better outcomes in high-TMB tumors. Our findings indicate that, in routine practice, bTMB may reflect tumor burden and aggressive disease biology rather than tumor immunogenicity. This study highlights the need for careful interpretation of liquid biopsy biomarkers in mCRC.

## 1. Introduction

Tumor mutational burden (TMB) has emerged as a potential biomarker of tumor biology, therapeutic responsiveness, and clinical outcomes across multiple solid tumors [1,2]. In colorectal cancer (CRC), TMB is strongly influenced by microsatellite instability (MSI) status, with MSI-high (MSI-H) and POLE-mutant tumors exhibiting markedly elevated mutation rates and increased immunogenicity [3,4,5]. These hypermutated subtypes represent a minority of metastatic CRC (mCRC), yet they have demonstrated substantial benefit from immune checkpoint inhibitors [2,6,7,8,9,10,11]. In contrast, the vast majority of mCRC cases are microsatellite-stable (MSS) and characterized by low TMB, limited immunogenicity, and poor responsiveness to immunotherapy [12,13].

Most available evidence regarding TMB in CRC is derived from tissue-based assays. Tissue TMB (tTMB) has been associated with distinct molecular phenotypes, immune microenvironment features, and, in some studies, improved survival in specific subgroups such as *RAS* mutant or MSS tumors [14,15,16,17,18]. However, tissue-based testing is limited by tumor availability, sampling bias, and the inability to capture spatial and temporal heterogeneity. Liquid biopsy approaches, particularly circulating tumor DNA (ctDNA) profiling, offer a minimally invasive alternative that enables real-time assessment of tumor genomics [19,20]. Blood-based TMB (bTMB) derived from ctDNA has shown promise as a biomarker in several malignancies, yet its clinical significance in mCRC is far less established [21,22,23].

Importantly, bTMB may reflect biological and clinical dimensions distinct from those captured by tTMB [24,25]. Because bTMB is influenced by both the number of detectable mutations and the amount of ctDNA shed into circulation, it may be affected by tumor burden, metastatic distribution, and disease aggressiveness. These factors are particularly relevant in real-world metastatic populations, where liquid biopsy is often performed in patients with progressive or treatment-resistant disease. As a result, the prognostic implications of bTMB in mCRC may differ substantially from those reported for tTMB in clinical trial settings.

To date, limited data exist on the prognostic value of bTMB in mCRC, and the relationship between bTMB, molecular subtypes, and clinical outcomes remains poorly understood. In this study, we analyzed a cohort of 255 patients with proficient mismatch repair system (pMMR)/MSS mCRC who underwent comprehensive genomic profiling using the FoundationOne^®^ Liquid assay [26]. We evaluated the distribution of bTMB, its association with clinicopathologic features, and its prognostic impact on overall survival (OS).

## 2. Materials and Methods

### 2.1. Study Design and Population

This was a monocentric, real-world observational study conducted in routine clinical practice. Consecutive adult patients (≥18 years) with mCRC who underwent liquid biopsy for comprehensive genomic profiling were eligible for inclusion, irrespective of treatment line or prior therapies. Patients with deficient MMR (dMMR)/MSI-high tumors were excluded. Patients were included if a FoundationOne^®^ Liquid CDx assay was successfully performed and if clinical follow-up data were available. Informed consent for genomic testing and data use was mandatory prior to sample collection, in accordance with institutional and ethical requirements.

### 2.2. Liquid Biopsy and Genomic Profiling

ctDNA analysis was performed using the FoundationOne^®^ Liquid CDx assay (Foundation Medicine, Inc., Cambridge, MA, USA), a clinically validated next-generation sequencing platform designed to detect genomic alterations. bTMB was derived by enumerating all coding variants—synonymous and non-synonymous—detected at or above a 0.5% allele frequency. Variants suspected to represent germline events were removed through a combination of population-level filtering and a dedicated somatic–germline discrimination algorithm. In addition, alterations classified as established or probable driver mutations were excluded to avoid inflating the mutational load. The final count of retained mutations was normalized to the size of the interrogated coding region (approximately 750 kb) and expressed as mutations per megabase. For the purpose of this study, the median bTMB value was used to define two groups: bTMB-low and bTMB-high. Importantly, bTMB results were not used to guide treatment decisions; all patients received standard-of-care therapies according to clinical guidelines.

### 2.3. Clinical Data Collection

Individual patient data were collected from electronic medical records (DxCare software, v8.2021.2.8, Dedalus, Antony, France). Treatment history, primary tumor, metastatic sites, *RAS/BRAF* status, and timing of liquid biopsy were recorded. Follow-up information was updated through routine clinical visits and medical documentation.

### 2.4. Endpoints

The primary endpoint was OS, defined as the time from the date of liquid biopsy to death from any cause. Patients alive at the time of analysis were censored at the date of last known follow-up. Secondary analyses included descriptive subgroup evaluations according to clinically relevant variables, including *RAS/BRAF* mutation status.

### 2.5. Statistical Analysis

Statistical analyses were performed using GraphPad Prism v22.021 software (MedCalc Software Ltd., Ostend, Belgium; https://www.medcalc.org; 2023). Continuous variables were summarized using medians and interquartile ranges, and categorical variables using frequencies and percentages. OS was estimated using the Kaplan–Meier method, and survival curves were compared using the log-rank test. Hazard ratios (HRs) and 95% confidence intervals (CIs) were calculated using Cox proportional hazards models. Subgroup analyses were exploratory and descriptive in nature. A two-sided *p*-value < 0.05 was considered statistically significant. Receiver operating characteristic (ROC) curve analysis with bootstrap resampling was performed to confirm the optimal threshold for bTMB in relation to the one-year survival rate.

## 3. Results

### 3.1. Patient Characteristics

**Figure 1:**
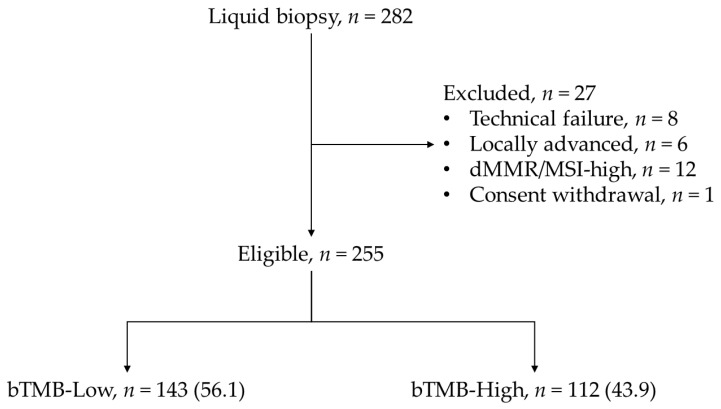
Study flowchart of patient inclusion and exclusion. Out of 282 patients who underwent ctDNA testing, 27 were excluded due to technical failure (*n* = 8), locally advanced disease (*n* = 6), dMMR/MSI-high tumors (*n* = 12) or withdrawal of consent (*n* = 1). The final cohort included 255 patients.

Out of 282 patients who underwent ctDNA testing, 27 were excluded due to technical failure (*n* = 8), locally advanced disease (*n* = 6), dMMR/MSI-high tumors (*n* = 12), or withdrawal of consent (*n* = 1). The final cohort included 255 patients with pMMR/MSS mCRC (Figure 1).

All patients underwent liquid biopsy using the FoundationOne^®^ Liquid CDx assay in routine clinical practice. Baseline demographic and clinical characteristics are summarized in Table 1.

The median age at the time of liquid biopsy was 65.0 years (range: 27.6 to 93.8), and 48.2% of patients were female. The majority of tumors were left-sided (68.6%), and liver metastases were present in 67.8% of cases. *RAS* mutations were identified in 54.5% of patients, and *BRAF^V600E^* mutations in 8.6%.

### 3.2. Distribution of Blood TMB

**Figure 2:**
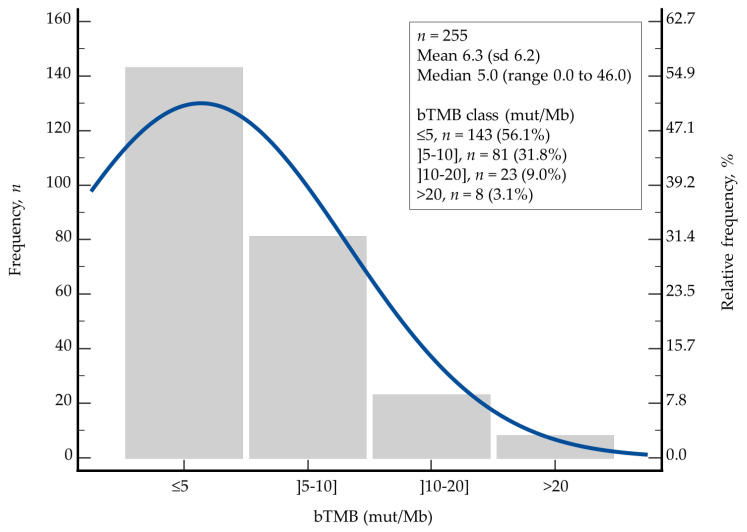
Distribution plot. Histogram of frequency (y-axis) and relative frequency (secondary y-axis) of blood TMB (mut/Mb) in 255 patients with pMMR/MSS mCRC. bTMB demonstrated a markedly heterogeneous distribution within the cohort. As a continuous variable, bTMB exhibited a mean of 6.3 mut/Mb (standard deviation (sd), 6.2) and a median of 5.0 mut/Mb (range, 0.0 to 46.0).

bTMB exhibited a right-skewed distribution across the study population. The mean bTMB was 6.3 mut/Mb with a standard deviation of 6.2, while the median value was 5.0 mut/Mb (range, 0.0 to 46.0). For descriptive purposes, bTMB was further categorized into four semi-quantitative classes: ≤5 mut/Mb (*n* = 143), >5–10 mut/Mb (*n* = 81), >10–20 mut/Mb (*n* = 23), and >20 mut/Mb (*n* = 8). This distribution highlights that most patients presented with low bTMB values (Figure 2).

Based on the median value, patients were classified into two groups: bTMB-low (bTMB ≤ 5, *n* = 143) and bTMB-high (bTMB > 5, *n* = 112). The proportion of patients in each group was 56.1% and 43.9%, respectively (Figure 1). The ROC curve yielded an area under the curve (AUC) of 0.69 (95% CI 0.63 to 0.75; *p* < 0.0001). The Youden index identified an optimal cutoff at >4 mut/Mb with sensitivity and specificity rates of 72.2% (95% CI 62.8 to 80.4) and 57.8% (95% CI 45.4 to 65.9).

Patient and tumor characteristics were well balanced between the bTMB-low and bTMB-high groups, with no significant differences observed across demographic, molecular, or metastatic variables (Table 1). The only notable distinction was the treatment setting, as patients with a high bTMB were more frequently treated in later-line therapy (*p* = 0.017).

The proportion of patients with a high ctDNA fraction (≥10%) was greater in the bTMB-high group than in the bTMB-low group (77.6% vs. 22.4%; *p* < 0.0001). The median bTMB was 3.5 (range: 0.0 to 13.0) and 8.0 (range: 1.0 to 46.0) in the ctDNA fraction-low and ctDNA fraction-high groups, respectively.

### 3.3. Overall Survival

**Figure 3:**
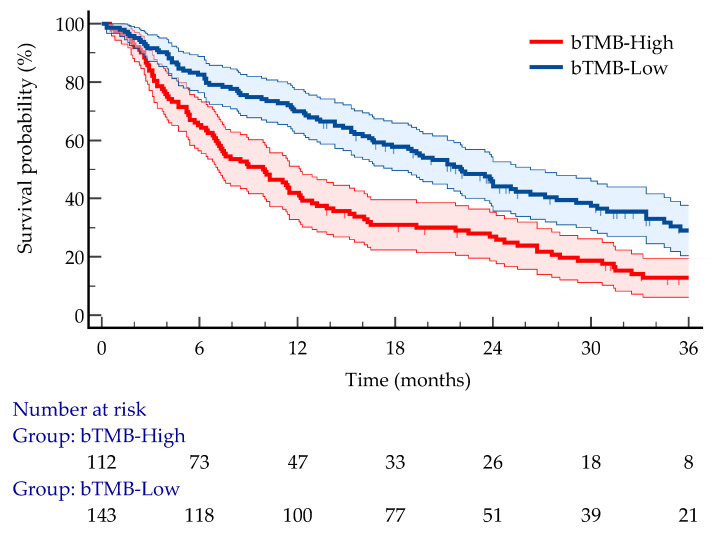
Kaplan–Meier curves for overall survival stratified by blood TMB (blue solid line, bTMB-low (≤5 mut/Mb); red solid line, bTMB-high (>5 mut/Mb)). The high-expression group demonstrated significantly poorer survival compared to the low-expression group (log-rank *p* < 0.0001; HR 1.88; 95% CI 1.39 to 2.53).

At a median follow-up of 36.8 months (95% CI 33.1 to 58.6), 191 (74.9%) deaths had occurred. The median OS for the entire cohort was 15.5 months (95% CI 12.4 to 19.5). Patients with bTMB-high tumors had significantly shorter OS compared with those with bTMB-low tumors. Median OS was 9.9 months (95% CI 7.1 to 12.1) in the bTMB-high group versus 22.1 months (95% CI 17.3 to 26.3) in the bTMB-low group (log-rank *p* < 0.0001). In univariable Cox analysis, high bTMB was associated with an HR of 1.88 for death (95% CI 1.39 to 2.53), indicating a significantly increased risk of mortality (Figure 3).

### 3.4. Sensitivity Analysis

**Figure 4:**
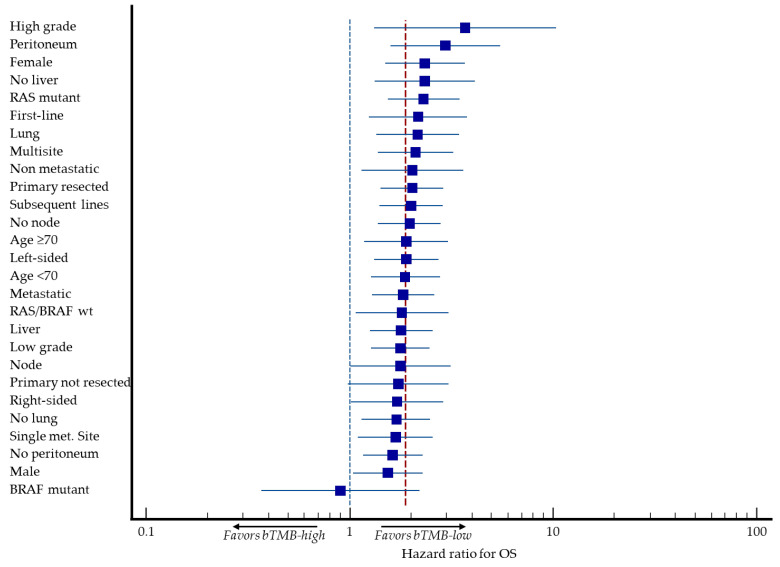
Forest plot showing hazard ratios for overall survival associated with a high bTMB (>5 mut/Mb) across predefined subgroups (e.g., age, sex, metastatic sites, treatment line), confirming consistent prognostic impact, except for *BRAF^V600E^* mutant tumors. The vertical blue dashed line represents the absence of prognostic impact (HR 1.0), the vertical red dashed line represents the overall prognostic impact of bTMB for OS in the whole population (HR 1.88), and the horizontal blue lines represent 95% CI. Logarithmic scale (*x*-axis).

Exploratory subgroup analyses were performed to evaluate the prognostic impact of bTMB across clinically relevant categories. The adverse prognostic effect of high bTMB was most pronounced in patients with high-grade tumors (HR 3.70; *p* = 0.013), peritoneal involvement (HR 2.95; *p* = 0.0006), a female gender (HR 2.35; *p* = 0.0002), extra-liver metastatic disease (HR 2.35; *p* = 0.003) and *RAS* mutant tumors (HR 2.32; *p* = 0.0001) (Table 2 and Figure 4).

No prognostic interaction was observed between bTMB and *BRAF^V600E^* mutant tumors (HR 0.90, 95% CI 0.37 to 2.21; *p* = 0.821), although these analyses were descriptive and not powered for formal interaction testing.

## 4. Discussion

In this real-world monocentric cohort of 255 patients with pMMR/MSS mCRC, we observed that elevated bTMB, measured using the FoundationOne^®^ Liquid CDx assay, was associated with significantly worse OS. This finding contrasts with several prior reports suggesting that high tTMB may be associated with favorable outcomes in selected colorectal cancer subgroups [18,27]. Our results highlight important biological and methodological differences between blood-based and tissue-based TMB assessments and suggest that bTMB may capture a distinct dimension of tumor biology in mCRC.

A key consideration when interpreting these findings is the fundamental difference between bTMB and tTMB. Tissue TMB primarily reflects the intrinsic mutational landscape of a sampled tumor region, whereas bTMB is influenced by both the number of detectable mutations and the amount of ctDNA shed into the bloodstream. High ctDNA shedding is strongly associated with tumor burden and metastatic spread [28,29,30,31]. As a result, in an unselected real-world metastatic population, bTMB may function as a surrogate marker of disease extent rather than a measure of tumor immunogenicity. This biological distinction may explain why high bTMB was associated with poor prognosis in our cohort of patients with pMMR/MSS tumors, whereas high tTMB has been linked to favorable outcomes in more selected populations, particularly those enriched for MSI-H or POLE-mutated tumors [15,16,17]. To further contextualize this finding, it is important to note that most studies reporting a favorable association between high TMB and survival have relied on *tissue*-based TMB, particularly in non-small cell lung cancer, where ctDNA shedding is generally lower and bTMB is less confounded by tumor burden. In contrast, in our unselected metastatic pMMR/MSS population, high bTMB likely reflects increased ctDNA release from extensive disease rather than intrinsic tumor immunogenicity. This biological and methodological distinction may therefore account for the inverse prognostic association observed in our cohort.

The distribution of bTMB values in our study further supports this interpretation. The median bTMB was 5 mut/Mb, consistent with the microsatellite-stable biology. Using the median as a cutoff likely separates patients with low ctDNA shedding from those with higher shedding, rather than distinguishing biologically hypermutated tumors from non-hypermutated ones. This is further supported by our finding that bTMB was strongly correlated with ctDNA fraction (*p* < 0.0001). Prior studies reporting favorable outcomes in high-TMB CRC often used higher absolute thresholds or focused on hypermutated subtypes. Thus, the prognostic meaning of “high TMB” differs substantially depending on the assay, cutoff, and population studied. Statistical considerations may have influenced the results. Dichotomizing a continuous variable such as TMB at the median may obscure non-linear relationships or threshold effects. It is plausible that only very high TMB values—typically associated with MSI-H or POLE-mutated tumors—confer favorable prognosis, whereas intermediate elevations reflect aggressive disease biology. In an MSS population, median-based dichotomization may therefore preferentially capture the latter group.

The clinical context in which liquid biopsies were obtained may also have contributed to the observed association. In routine practice, liquid biopsy is frequently performed in patients with progressive disease, limited tissue availability, or complex therapeutic decision points. These patients may have a higher tumor burden or more aggressive disease, potentially enriching the bTMB-high group for individuals with poor prognosis. This contrasts with clinical trial settings, where tissue samples are typically collected at baseline in more homogeneous and less heavily pretreated populations.

Interestingly, the adverse prognostic effect of high bTMB was most pronounced in patients with *RAS* mutant tumors. *RAS* mutant mCRC is characterized by distinct biological features, including higher metastatic potential and resistance to anti-epidermal growth factor receptor monoclonal antibodies [32,33,34,35,36]. In this subgroup, high bTMB may identify tumors with particularly aggressive clonal evolution or extensive metastatic dissemination. This observation underscores that bTMB and tTMB are not interchangeable biomarkers and may reflect different aspects of tumor biology depending on the molecular context.

Technical aspects of the FoundationOne^®^Liquid CDx assay may also influence bTMB interpretation [26]. Blood-based TMB estimation requires a minimum ctDNA fraction, potentially excluding patients with a low tumor burden or indolent disease from evaluable analyses. Conversely, patients with high ctDNA levels—often those with more advanced disease—are more likely to have sufficient detectable mutations to yield higher bTMB values.

Yeh et al. showed that acquired increases in plasma TMB after targeted therapy in patients with MSS mCRC do not reflect true hypermutation within tumor sites but instead arise from the aggregate shedding of numerous highly subclonal alterations from multiple progressing lesions [37]. This supports the interpretation that elevated bTMB often reflects ctDNA shedding dynamics rather than a biologically meaningful increase in intratumoral TMB, helping explain why such patients do not benefit from immune checkpoint blockade.

This study has several limitations that should be acknowledged. First, its monocentric and real-world design may introduce selection biases inherent to routine clinical practice. Liquid biopsies were performed at the discretion of treating physicians, often in the context of disease progression or therapeutic decision-making, which may have enriched the cohort for patients with more aggressive or treatment-resistant disease. Second, although the sample size of 255 patients is substantial for a single-center liquid biopsy study in mCRC, subgroup analyses—particularly those stratified by RAS status—were exploratory and not powered for definitive conclusions. Third, bTMB was assessed using the FoundationOne^®^Liquid CDx assay, which, like all ctDNA-based platforms, is influenced by ctDNA shedding and tumor burden. As a result, bTMB may reflect both genomic complexity and disease extent, making it difficult to disentangle biological from clinical determinants of prognosis. The use of the median bTMB value to define high and low groups, while pragmatic, may not correspond to biologically meaningful thresholds and may obscure non-linear or threshold-dependent effects. In addition, although modeling bTMB as a continuous or transformed variable would be preferable given its right-skewed distribution, the limited sample size and event count in our cohort resulted in unstable estimates; therefore, dichotomization at the median was used as the most statistically robust approach available. And, when interpreting the association between bTMB and OS, it is important to acknowledge the potential influence of confounding factors such as treatment line, treatment-related factors such as the use of biomarker-directed therapies, and ctDNA fraction. Although multivariable Cox modelling would have been the preferred approach to adjust for these variables, the limited sample size and number of events in our cohort resulted in unstable or non-convergent estimates when these covariates were included. For this reason, we restricted our analyses to univariable comparisons, and the observed associations should therefore be considered exploratory. This limitation is consistent with the broader challenge that bTMB in real-world metastatic populations may reflect disease burden and ctDNA shedding rather than intrinsic tumor biology, reinforcing the need for cautious interpretation. Finally, although OS was rigorously defined from the date of liquid biopsy, residual confounding cannot be excluded, as real-world datasets lack the uniform treatment protocols and standardized follow-up of prospective clinical trials.

Despite these limitations, the study provides valuable insights into the prognostic significance of bTMB in mCRC and highlights important differences between blood-based and tissue-based TMB assessments.

## 5. Conclusions

In this real-world cohort of patients with pMMR/MSS mCRC undergoing liquid comprehensive genomic profiling, elevated bTMB was independently associated with inferior OS, particularly in the subgroup of *RAS* mutant tumors. These findings contrast with prior reports suggesting favorable outcomes in high-TMB colorectal cancer and underscore that blood-based TMB reflects a distinct biological and clinical construct compared with tissue-derived TMB.

Our results highlight the need for context-specific interpretation of liquid biopsy biomarkers and support further prospective studies integrating bTMB with ctDNA fraction and tumor burden metrics to refine its prognostic and predictive utility in mCRC.

## Figures and Tables

**Table 1:** Patient and tumor characteristics, *n* (%).

Covariate	All Patients	Blood TMB-Low	Blood TMB-High	*p*-Value
No. of patients	255	143	112	
Age, years				0.102
<70	160 (62.7)	96 (67.1)	64 (57.1)	
≥70	95 (37.3)	47 (32.9)	48 (42.9)	
Sex				0.796
Female	123 (48.2)	70 (49.0)	53 (47.3)	
Male	132 (51.8)	73 (51.0)	59 (52.7)	
*RAS*/*BRAF* status				0.446
Wild-type	94 (36.9)	51 (35.7)	43 (38.4)	
*RAS* mutant	139 (54.5)	82 (57.3)	57 (50.9)	
*BRAFV600E* mutant	22 (8.6)	10 (7.0)	12 (10.7)	
Tumor sidedness				0.479
Right-sided	76 (29.8)	39 (27.3)	37 (33.0)	
Left-sided	175 (68.6)	101 (70.6)	74 (66.1)	
Both	4 (1.6)	3 (2.1)	1 (0.9)	
Grading				0.367
Low	201 (88.2)	108 (86.4)	93 (90.3)	
High	27 (11.8)	17 (13.6)	10 (9.7)	
Missing	27	18	9	
Initial stage				0.537
Non-metastatic	78 (30.6)	46 (32.2)	32 (28.6)	
Metastatic	177 (69.4)	97 (67.8)	80 (71.4)	
Primary resection				0.151
No	78 (30.6)	49 (34.3)	29 (25.9)	
Yes	177 (69.4)	94 (65.7)	83 (74.1)	
No. of met. Sites				0.182
1	135 (52.9)	81 (56.6)	54 (48.2)	
>1	120 (47.1)	62 (43.4)	58 (51.8)	
Liver involvement				0.059
No	82 (32.2)	53 (37.1)	29 (25.9)	
Yes	173 (67.8)	90 (62.9)	83 (74.1)	
Lung involvement				0.107
No	162 (63.5)	97 (67.8)	65 (58.0)	
Yes	93 (36.5)	46 (32.2)	47 (42.0)	
Node involvement				0.401
No	191 (74.9)	110 (76.9)	81 (72.3)	
Yes	64 (25.1)	33 (23.1)	31 (27.7)	
Peritoneal involvement				0.513
No	186 (72.9)	102 (71.3)	84 (75.0)	
Yes	69 (27.1)	41 (28.7)	28 (25.0)	
Treatment setting				0.017
First-line	96 (37.6)	54 (37.8)	42 (37.5)	
Second-line	57 (22.4)	40 (28.0)	17 (15.2)	
Third-line	62 (24.3)	34 (23.8)	28 (25.0)	
Subsequent lines	40 (15.7)	15 (10.5)	25 (22.3)	

**Table 2:** Univariate analysis for OS in bTMB-Low/bTMB-High subgroups.

Covariates	Class	*n*	Events	Median OS, Months	HR (95% CI)	*p*-Value
Age	<70	96/64	66/53	23.5/9.9	1.88 (1.27–2.78)	0.002
	≥70	47/48	30/42	21.2/9.0	1.90 (1.18–3.05)	0.008
Sex	Female	70/53	42/45	24.0/7.6	2.35 (1.50–3.70)	0.0002
	Male	73/59	54/50	19.0/11/5	1.55 (1.04–2.30)	0.032
*RAS*/*BRAF*	Wild-type	51/43	28/31	27.9/16.3	1.81 (1.07–3.07)	0.027
	*RAS* mutant	82/57	59/53	20.3/7.9	2.32 (1.54–3.49)	0.0001
	*BRAF* mutant	10/22	9/11	6.6/7.5	0.90 (0.37–2.21)	0.821
Sidedness	Right-sided	39/37	25/33	22.1/11.5	1.72 (1.02–2.89)	0.043
	Left-sided	101/74	69/61	21.6/9.0	1.90 (1.32–2.75)	0.0006
Grading	Low	108/93	72/76	22.3/10.3	1.78 (1.27–2.48)	0.0008
	High	17/10	11/10	21.6/8.9	3.70 (1.32–10.33)	0.013
Initial stage	Non-metastatic	46/32	24/27	44.5/12/2	2.04 (1.15–3.61)	0.015
	Metastatic	97/80	72/68	21.2/7.6	1.83 (1.29–2.60)	0.0008
Primary resection	No	49/29	37/23	17.8/6.9	1.74 (0.98–3.08)	0.059
	Yes	94/83	59/72	24.0/11.1	2.03 (1.42–2.89)	0.0001
No. of sites	1	81/54	54/44	22.1/11.8	1.69 (1.10–2.57)	0.015
	>1	62/58	42/51	21.6/7.9	2.12 (1.38–3.24)	0.0006
Liver involvement	No	53/29	36/27	17.3/9.9	2.35 (1.33–4.13)	0.003
	Yes	90/83	60/68	23.8/10.0	1.80 (1.26–2.57)	0.001
Lung involvement	No	97/65	63/52	22.1/10.3	1.70 (1.15–2.50)	0.008
	Yes	46/47	33/43	22.0/8.5	2.16 (1.35–3.46)	0.001
Node involvement	No	110/81	73/68	22.1/8.9	1.97 (1.38–2.80)	0.0002
	Yes	33/31	23/27	19.7/10.3	1.78 (1.01–3.15)	0.048
Peritoneal involvement	No	102/84	69/70	22.1/11.4	1.63 (1.16–2.30)	0.005
	Yes	41/28	27/25	21.6/5.4	2.95 (1.59–5.51)	0.0006
Treatment setting	First-line	54/42	23/30	39.8/22.5	2.17 (1.24–3.79)	0.006
	Subsequent lines	89/70	73/65	16.0/6.8	2.00 (1.40–2.88)	0.0002

## Data Availability

The data supporting the findings of this study are available on request from the corresponding author. The data are not publicly available due to their containing information that could compromise the privacy of research participants.

## Abbreviations

The following abbreviations are used in this manuscript:

TMB	Tumor mutational burden
CRC	Colorectal cancer
MSI	Microsatellite instability
mCRC	Metastatic colorectal cancer
MSS	Microsatellite stable
tTMB	Tumor-based tumor mutational burden
ctDNA	Circulating tumor DNA
pMMR	Proficient mismatch repair system
OS	Overall survival
dMMR	Deficient mismatch repair system
HR	Hazard ratio
CI	Confidence interval
ROC	Receiver operating characteristic
AUC	Area under curve
KRAS	V-Ki-ras2 Kirsten rat sarcoma viral oncogene homolog
NRAS	neuroblastoma RAS viral oncogene homolog
*BRAF*	proto-oncogene B-Raf and v-Raf murine sarcoma viral oncogene homolog B

## References

[1] Chalmers Z.R., Connelly C.F., Fabrizio D., Gay L., Ali S.M., Ennis R., Schrock A., Campbell B., Shlien A., Chmielecki J. (2017). Analysis of 100,000 human cancer genomes reveals the landscape of tumor mutational burden. Genome Med..

[2] Marabelle A., Fakih M., Lopez J., Shah M., Shapira-Frommer R., Nakagawa K., Chung H.C., Kindler H.L., Lopez-Martin J.A., Miller W.H. (2020). Association of tumour mutational burden with outcomes in patients with advanced solid tumours treated with pembrolizumab: Prospective biomarker analysis of the multicohort, open-label, phase 2 KEYNOTE-158 study. Lancet Oncol..

[3] Tougeron D., Fauquembergue E., Rouquette A., Le Pessot F., Sesboüé R., Laurent M., Berthet P., Mauillon J., Di Fiore F., Sabourin J.C. (2009). Tumor-infiltrating lymphocytes in colorectal cancers with microsatellite instability are correlated with the number and spectrum of frameshift mutations. Mod. Pathol..

[4] Fabrizio D.A., George T.J., Dunne R.F., Frampton G., Sun J., Gowen K., Kennedy M., Greenbowe J., Schrock A.B., Hezel A.F. (2018). Beyond microsatellite testing: Assessment of tumor mutational burden identifies subsets of colorectal cancer who may respond to immune checkpoint inhibition. J. Gastrointest. Oncol..

[5] Rousseau B., Bieche I., Pasmant E., Hamzaoui N., Leulliot N., Michon L., de Reynies A., Attignon V., Foote M.B., Masliah-Planchon J. (2022). PD-1 blockade in solid tumors with defects in polymerase epsilon. Cancer Discov..

[6] Ambrosini M., Rousseau B., Manca P., Artz O., Marabelle A., André T., Maddalena G., Mazzoli G., Intini R., Cohen R. (2024). Immune checkpoint inhibitors for POLE or POLD1 proofreading-deficient metastatic colorectal cancer. Ann. Oncol..

[7] Schrock A.B., Ouyang C., Sandhu J., Sokol E., Jin D., Ross J.S., Miller V.A., Lim D., Amanam I., Chao J. (2019). Tumor mutational burden is predictive of response to immune checkpoint inhibitors in MSI-high metastatic colorectal cancer. Ann. Oncol..

[8] André T., Shiu K.K., Kim T.W., Jensen B.V., Jensen L.H., Punt C., Smith D., Garcia-Carbonero R., Benavides M., Gibbs P. (2020). KEYNOTE-177 Investigators. Pembrolizumab in microsatellite-instability-high advanced colorectal cancer. N. Engl. J. Med..

[9] André T., Shiu K.K., Kim T.W., Jensen B.V., Jensen L.H., Punt C.J.A., Smith D., Garcia-Carbonero R., Alcaide-Garcia J., Gibbs P. (2025). Pembrolizumab versus chemotherapy in microsatellite instability-high or mismatch repair-deficient metastatic colorectal cancer: 5-year follow-up from the randomized phase III KEYNOTE-177 study. Ann. Oncol..

[10] Andre T., Elez E., Van Cutsem E., Jensen L.H., Bennouna J., Mendez G., Schenker M., de la Fouchardiere C., Limon M.L., Yoshino T. (2024). CheckMate 8HW Investigators. Nivolumab plus ipilimumab in microsatellite-instability-high metastatic colorectal cancer. N. Engl. J. Med..

[11] André T., Elez E., Lenz H.J., Jensen L.H., Touchefeu Y., Van Cutsem E., Garcia-Carbonero R., Tougeron D., Mendez G.A., Schenker M. (2025). Nivolumab plus ipilimumab versus nivolumab in microsatellite instability-high metastatic colorectal cancer (CheckMate 8HW): A randomised, open-label, phase 3 trial. Lancet.

[12] Huyghe N., Benidovskaya E., Stevens P., Van den Eynde M. (2022). Biomarkers of response and resistance to immunotherapy in microsatellite stable colorectal cancer: Toward a new personalized medicine. Cancers.

[13] Gandini A., Puglisi S., Pirrone C., Martelli V., Catalano F., Nardin S., Seeber A., Puccini A., Sciallero S. (2023). The role of immunotherapy in microsatellites stable metastatic colorectal cancer: State of the art and future perspectives. Front. Oncol..

[14] Merino D.M., McShane L.M., Fabrizio D., Funari V., Chen S.J., White J.R., Wenz P., Baden J., Barrett J.C., Chaudhary R. (2020). TMB Harmonization Consortium. Establishing guidelines to harmonize tumor mutational burden (TMB): In silico assessment of variation in TMB quantification across diagnostic platforms: Phase I of the Friends of Cancer Research TMB Harmonization Project. J. Immunother. Cancer.

[15] Salem M.E., Puccini A., Grothey A., Raghavan D., Goldberg R.M., Xiu J., Korn W.M., Weinberg B.A., Hwang J.J., Shields A.F. (2018). Landscape of tumor mutation load, mismatch repair deficiency, and PD-L1 expression in a large patient cohort of gastrointestinal cancers. Mol. Cancer Res..

[16] Picard E., Verschoor C.P., Ma G.W., Pawelec G. (2020). Relationships between immune landscapes, genetic subtypes and responses to immunotherapy in colorectal cancer. Front. Immunol..

[17] Lee D.W., Han S.W., Bae J.M., Jang H., Han H., Kim H., Bang D., Jeong S.Y., Park K.J., Kang G.H. (2019). Tumor mutation burden and prognosis in patients with colorectal cancer treated with adjuvant fluoropyrimidine and oxaliplatin. Clin. Cancer Res..

[18] Di Mauro A., Santorsola M., Savarese G., Sirica R., Ianniello M., Cossu A.M., Ceccarelli A., Sabbatino F., Bocchetti M., Carratù A.C. (2024). High tumor mutational burden assessed through next-generation sequencing predicts favorable survival in microsatellite stable metastatic colon cancer patients. J. Transl. Med..

[19] Wan J.C.M., Massie C., Garcia-Corbacho J., Mouliere F., Brenton J.D., Caldas C., Pacey S., Baird R., Rosenfeld N. (2017). Liquid biopsies come of age: Towards implementation of circulating tumour DNA. Nat. Rev. Cancer.

[20] Siravegna G., Marsoni S., Siena S., Bardelli A. (2017). Integrating liquid biopsies into the management of cancer. Nat. Rev. Clin. Oncol..

[21] Gandara D.R., Paul S.M., Kowanetz M., Schleifman E., Zou W., Li Y., Rittmeyer A., Fehrenbacher L., Otto G., Malboeuf C. (2018). Blood-based tumor mutational burden as a predictor of clinical benefit in non-small-cell lung cancer patients treated with atezolizumab. Nat. Med..

[22] Wang Z., Duan J., Cai S., Han M., Dong H., Zhao J., Zhu B., Wang S., Zhuo M., Sun J. (2019). Assessment of blood tumor mutational burden as a potential biomarker for immunotherapy in patients with non-small cell lung cancer with use of a next-generation sequencing cancer gene panel. JAMA Oncol..

[23] Chae Y.K., Davis A.A., Agte S., Pan A., Simon N.I., Iams W.T., Cruz M.R., Tamragouri K., Rhee K., Mohindra N. (2019). Clinical implications of circulating tumor DNA tumor mutational burden (ctDNA TMB) in non-small cell lung cancer. Oncologist.

[24] Fridland S., Choi J., Nam M., Schellenberg S.J., Kim E., Lee G., Yoon N., Chae Y.K. (2021). Assessing tumor heterogeneity: Integrating tissue and circulating tumor DNA (ctDNA) analysis in the era of immune-oncology—Blood TMB is not the same as tissue TMB. J. Immunother. Cancer.

[25] Sturgill E.G., Misch A., Jones C.C., Luckett D., Fu X., Schlauch D., Jones S.F., Burris H.A., Spigel D.R., McKenzie A.J. (2022). Discordance in tumor mutation burden from blood and tissue affects association with response to immune checkpoint inhibition in real-world settings. Oncologist.

[26] Woodhouse R., Li M., Hughes J., Delfosse D., Skoletsky J., Ma P., Meng W., Dewal N., Milbury C., Clark T. (2020). Clinical and analytical validation of FoundationOne Liquid CDx, a novel 324-gene cfDNA-based comprehensive genomic profiling assay for cancers of solid tumor origin. PLoS ONE.

[27] Wang J., Song J., Liu Z., Zhang T., Liu Y. (2022). High tumor mutation burden indicates better prognosis in colorectal cancer patients with KRAS mutations. Front. Oncol..

[28] Bettegowda C., Sausen M., Leary R.J., Kinde I., Wang Y., Agrawal N., Bartlett B.R., Wang H., Luber B., Alani R.M. (2014). Detection of circulating tumor DNA in early- and late-stage human malignancies. Sci. Transl. Med..

[29] Diehl F., Schmidt K., Choti M.A., Romans K., Goodman S., Li M., Thornton K., Agrawal N., Sokoll L., Szabo S.A. (2008). Circulating mutant DNA to assess tumor dynamics. Nat. Med..

[30] Thierry A.R., Mouliere F., El Messaoudi S., Mollevi C., Lopez-Crapez E., Rolet F., Gillet B., Gongora C., Dechelotte P., Robert B. (2014). Clinical validation of the detection of KRAS and BRAF mutations from circulating tumor DNA. Nat. Med..

[31] Newman A.M., Bratman S.V., To J., Wynne J.F., Eclov N.C., Modlin L.A., Liu C.L., Neal J.W., Wakelee H.A., Merritt R.E. (2014). An ultrasensitive method for quantitating circulating tumor DNA with broad patient coverage. Nat. Med..

[32] Lièvre A., Bachet J.B., Le Corre D., Boige V., Landi B., Emile J.F., Côté J.F., Tomasic G., Penna C., Ducreux M. (2006). KRAS mutation status is predictive of response to cetuximab therapy in colorectal cancer. Cancer Res..

[33] Amado R.G., Wolf M., Peeters M., Van Cutsem E., Siena S., Freeman D.J., Juan T., Sikorski R., Suggs S., Radinsky R. (2008). Wild-type KRAS is required for panitumumab efficacy in patients with metastatic colorectal cancer. J. Clin. Oncol..

[34] Karapetis C.S., Khambata-Ford S., Jonker D.J., O’Callaghan C.J., Tu D., Tebbutt N.C., Simes R.J., Chalchal H., Shapiro J.D., Robitaille S. (2008). K-ras mutations and benefit from cetuximab in advanced colorectal cancer. N. Engl. J. Med..

[35] Yaeger R., Cowell E., Chou J.F., Gewirtz A.N., Borsu L., Vakiani E., Solit D.B., Rosen N., Capanu M., Ladanyi M. (2015). RAS mutations affect pattern of metastatic spread and increase propensity for brain metastasis in colorectal cancer. Cancer.

[36] Cercek A., Braghiroli M.I., Chou J.F., Hechtman J.F., Kemeny N., Saltz L., Capanu M., Yaeger R. (2017). Clinical features and outcomes of patients with colorectal cancers harboring NRAS mutations. Clin. Cancer Res..

[37] Yeh C., Artz O., Zhang H., Karnoub E.R., Ntiamoah P., Weipert C., Walch H., Harrold E., Keane F., Chalasani S. (2025). Acquired high tumor mutational burden and activity of immunotherapy after targeted therapy in microsatellite stable colorectal cancer. Clin. Cancer Res..

